# Clinical Outcomes and Prognostic Factors Following Routine CT-Based Image-Guided Adaptive Brachytherapy for Cervical Cancer: A Retrospective Cohort Study

**DOI:** 10.3390/cancers18172805

**Published:** 2026-08-28

**Authors:** Pooriwat Muangwong, Ekkasit Tharavichitkul, Somvilai Chakrabandhu, Pitchayaponne Klunklin, Wimrak Onchan, Bongkot Jia-Mahasap, Piyapasara Toapichattrakul, Wannapha Nobnop, Anirut Watcharawipha, Razvan M. Galalae, Imjai Chitapanarux

**Affiliations:** 1 Division of Radiation Oncology, Department of Radiology, Faculty of Medicine, Chiang Mai University, Chiang Mai 50200, Thailand; pooriwat.m@cmu.ac.th (P.M.); pitchayaponne.kl@cmu.ac.th (P.K.); wimrak.o@cmu.ac.th (W.O.); bongkot.su@cmu.ac.th (B.J.-M.); piyapasara.t@cmu.ac.th (P.T.); wannapha.n@cmu.ac.th (W.N.); anirut.watch@cmu.ac.th (A.W.);; 2 Faculty of Medicine, Christian-Albrechts-University, 24118 Kiel, Germany; razvan.galalae@klinikum-bremerhaven.de; 3 Radiation Oncology Department, Klinikum Bremerhaven, 27574 Bremerhaven, Germany

**Keywords:** cervical cancer, CT-based IGABT, image-guided brachytherapy, local control, overall survival

## Abstract

**Background/Objectives**: Magnetic resonance imaging (MRI)-based image-guided adaptive brachytherapy (IGABT) is the preferred standard for cervical cancer, but access remains limited in many settings. Computed tomography (CT)-based IGABT provides a practical alternative. We evaluated clinical outcomes and prognostic factors following its routine implementation. **Methods:** This retrospective cohort study included patients with International Federation of Gynecology and Obstetrics (FIGO) 2018 stage I–IVA cervical carcinoma treated with definitive radiotherapy incorporating CT-based IGABT from January 2019 to December 2021. Patients received pelvic external beam radiotherapy (45–50.4 Gy in 23–28 fractions), with or without concurrent platinum-based chemotherapy, followed by four high-dose-rate brachytherapy fractions. Local control and overall survival were estimated using Kaplan–Meier analysis, and prognostic factors were evaluated using Cox regression. **Results:** Among 237 patients, 56.9% had FIGO stage III–IV disease, and hybrid intracavitary/interstitial brachytherapy was used in 30.2% of fractions. The mean high-risk clinical target volume D90 (HR-CTV D90) was 85.4 ± 2.7 Gy EQD_2_. Median follow-up was 59.9 months for local control and 71.1 months for overall survival. The 5-year local control and overall survival rates were 84.4% (95% CI, 79.1–88.5%) and 62.9% (95% CI, 56.3–68.8%), respectively. Squamous histology was independently associated with improved local control and overall survival. HR-CTV D90 ≥ 85 Gy EQD_2_ was associated with improved local control, whereas ≥4 chemotherapy cycles and overall treatment time ≤ 56 days were associated with improved overall survival. **Conclusions:** Routine CT-based IGABT achieved clinically meaningful outcomes despite the high proportion of advanced-stage disease. Achieving HR-CTV D90 ≥ 85 Gy EQD_2_, delivering ≥4 chemotherapy cycles, and completing treatment within 56 days were associated with improved outcomes.

## Simple Summary

MRI-guided brachytherapy provides highly accurate treatment for cervical cancer but is not routinely available in many hospitals. CT-based brachytherapy offers a more accessible alternative, although long-term clinical evidence remains limited. We evaluated 237 patients with cervical cancer treated using routine CT-based image-guided adaptive brachytherapy. Despite more than half of the patients having stage III–IV disease, the 5-year local control and overall survival rates were 84.4% and 62.9%, respectively. Delivering an adequate brachytherapy dose to the tumor was associated with improved local control, while receiving at least four chemotherapy cycles and completing treatment within 56 days were associated with improved overall survival. These findings suggest that a standardized CT-based brachytherapy program can provide clinically meaningful outcomes in centers where routine MRI access is limited.

## 1. Introduction

Cervical cancer remains a major public health concern in Thailand and worldwide. In 2022, approximately 600,000 new cases were diagnosed globally, while Thailand recorded 8662 new cases and 4576 deaths in the same year [1].

Cervical cancer can be treated with surgery, radiotherapy, and systemic therapy, depending on disease stage and patient factors. For patients undergoing definitive radiotherapy, the standard approach consists of external beam radiotherapy, with concurrent cisplatin-based chemotherapy for those with locally advanced disease, followed by brachytherapy [2,3].

Brachytherapy is an essential component of this definitive regimen because it enables delivery of a high radiation dose to the primary tumor while limiting exposure to adjacent organs at risk. Consequently, the delivery of high-quality brachytherapy is a critical determinant of local control and survival outcomes [4].

The development of image-guided adaptive brachytherapy (IGABT) has substantially improved the precision of target delineation and dose delivery. Magnetic resonance imaging (MRI)-guided IGABT provides superior soft-tissue contrast, enabling individualized target definition and adaptive dose optimization. Early clinical experience demonstrated the feasibility and clinical impact of MRI-guided IGABT [5]. Subsequent large multicenter studies, including Retro-EMBRACE and the prospective EMBRACE-I cohort, reported high rates of long-term local control and overall survival with acceptable rates of severe morbidity, establishing MRI-guided IGABT as a benchmark standard in well-resourced settings [6,7].

However, access to MRI during brachytherapy remains limited in many regions because of limited MRI availability and logistical constraints associated with obtaining MRI during brachytherapy procedures, with recent practice-pattern data identifying MRI access as a major barrier to routine implementation [8,9]. In this context, computed tomography (CT)-based IGABT has emerged as a practical alternative. CT-based planning enables three-dimensional dose optimization and volumetric evaluation of target coverage and organs at risk constraints. Although CT has inherent limitations in soft-tissue contrast, international consensus guidelines have been developed to standardize CT-based contouring, and clinical studies have reported promising outcomes with this approach [10,11,12,13].

At our institution, CT-based IGABT was introduced in 2008 and subsequently developed through the incorporation of CT-based treatment planning and transabdominal ultrasound guidance. Our previous studies demonstrated the feasibility of these image-guided approaches in routine clinical practice [14,15]. In December 2018, the installation of a dedicated CT scanner (Alexion, Canon Medical Systems, Japan) within the brachytherapy unit enabled the full integration of CT-based IGABT into our routine clinical workflow. Outcomes from the earlier implementation period between 2008 and 2018 have been reported, with a 4-year overall survival rate of 69.1% [16].

The present study evaluates clinical outcomes and prognostic factors in patients treated with definitive radiotherapy incorporating routine CT-based IGABT between January 2019 and December 2021, following the implementation of dedicated CT-based brachytherapy in our division.

## 2. Materials and Methods

### 2.1. Study Design and Population

This retrospective cohort study evaluated clinical outcomes following the implementation of routine CT-based IGABT in the Division of Radiation Oncology, Faculty of Medicine, Chiang Mai University. The study was reviewed by the Research Ethics Committee of the Faculty of Medicine, Chiang Mai University and was certified as exempt from ethical review (RAD-2568-0136). The requirement for written informed consent was waived by the Research Ethics Committee because the study involved retrospective analysis of anonymized clinical data. The study was conducted in accordance with the Declaration of Helsinki.

All consecutive patients with cervical cancer treated with curative-intent external beam radiotherapy followed by CT-guided brachytherapy between January 2019 and December 2021 were included.

Inclusion criteria were: (1) International Federation of Gynecology and Obstetrics (FIGO) 2018 stage I–IVA cervical carcinoma; (2) treatment with definitive radiotherapy as the primary modality; (3) histology of squamous cell carcinoma, adenocarcinoma, or adenosquamous carcinoma; and (4) availability of complete clinical, treatment, and dosimetric data.

Exclusion criteria included: (1) other histological subtypes; (2) evidence of distant metastatic disease at diagnosis; (3) treatment with non-standard external beam radiotherapy schedules; (4) incomplete medical records or absence of post-treatment follow-up data; or (5) prior pelvic radiotherapy or concurrent malignancies.

### 2.2. Data Collection

Clinical, treatment, dosimetric, and outcome data were extracted from institutional electronic medical records. Vital status and dates of death were verified using the hospital cancer registry. Local control was defined as the time from the start of radiotherapy to the date of documented local recurrence, with patients without local recurrence censored at their last documented disease assessment. Overall survival was defined as the time from the start of radiotherapy to death from any cause, with surviving patients censored at the date they were last known to be alive.

### 2.3. Treatments and Follow-Up

External beam radiotherapy was delivered to the pelvis at a dose of 45–50.4 Gy in 23–28 fractions, at 1.8–2.0 Gy per fraction, using either a four-field box technique or intensity-modulated radiation therapy (IMRT). Dose schedules included 45 Gy in 25 fractions, 46 Gy in 23 fractions, 50 Gy in 25 fractions, and 50.4 Gy in 28 fractions, which varied during the study period according to institutional practice at the time. The pelvic clinical target volume included the cervix, entire uterus, parametria, upper vagina, and regional pelvic lymph nodes for both conventional radiotherapy and IMRT. The treatment technique was selected at the discretion of the treating physician based on clinical requirements. Extended-field radiotherapy was delivered to patients with para-aortic nodal involvement and to those with common iliac nodal involvement or three or more involved pelvic lymph nodes. Involved lymph nodes received additional irradiation using either a simultaneous integrated boost or a sequential boost. Concurrent weekly cisplatin 40 mg/m^2^ or carboplatin AUC 2 was administered when clinically indicated. Induction chemotherapy was used selectively in patients with prolonged waiting times for radiotherapy, particularly during the COVID-19 pandemic.

High-dose-rate CT-based IGABT was delivered in four fractions, with a prescribed dose of 7 Gy per fraction, following external beam radiotherapy. Intracavitary applicators were used either alone or in combination with interstitial needles (hybrid intracavitary/interstitial [IC/IS] technique), depending on tumor extent and anatomical geometry.

Applicator insertion was performed under transabdominal ultrasound guidance using tandem–ovoid, tandem–ring, or tandem–cylinder applicators. A hybrid IC/IS approach was considered for patients with residual parametrial involvement or asymmetric disease, based on physician judgment. The brachytherapy approach was assessed at each fraction and could be adapted according to tumor response and anatomical findings.

After each applicator insertion, CT-based contouring of the high-risk clinical target volume (HR-CTV) and organs at risk, including the bladder, rectum, and sigmoid colon, was performed in accordance with established international recommendations for three-dimensional image-guided cervical brachytherapy and CT-based contouring guidance [10,17,18,19]. The HR-CTV encompassed the entire cervix and residual macroscopic tumor at the time of brachytherapy. The intermediate-risk clinical target volume (IR-CTV) was not routinely delineated. Treatment planning and optimization were based on dose–volume histogram parameters.

External beam radiotherapy and brachytherapy doses were summed using the equivalent dose in 2-Gy fractions (EQD_2_) concept, applying α/β ratios of 10 Gy for tumor tissue and 3 Gy for late-responding normal tissues. Brachytherapy plans were optimized to achieve adequate target coverage while respecting organs-at-risk dose constraints. A cumulative HR-CTV dose covering 90% of the target volume (HR-CTV D90) was pursued, with further dose escalation when feasible. If an HR-CTV D90 of 85 Gy EQD_2_ could not be achieved, a cumulative dose of ≥80 Gy EQD_2_ was considered acceptable.

After completion of definitive radiotherapy, patients were scheduled for follow-up at six weeks, then every three months for the first year, every four months in the second year, every six months in the third and fourth years, and annually thereafter. Treatment response was assessed at the six-week follow-up based on clinical examination, with imaging performed when clinically indicated.

### 2.4. Statistical Analysis

Descriptive statistics were used to summarize baseline and treatment characteristics. Local control and overall survival were estimated using the Kaplan–Meier method, and differences between groups were assessed using the log-rank test. As an additional analysis, the cumulative incidence of local recurrence was estimated with death without prior local recurrence treated as a competing event. Prognostic factors were evaluated using univariable and multivariable Cox proportional hazards regression, reporting hazard ratios (HRs) with 95% confidence intervals (CIs). Variables were selected a priori for inclusion in the multivariable models based on clinical relevance and included age group, stage grouping, histology, tumor size, HR-CTV dose, chemotherapy exposure, overall treatment time, nodal status, external beam radiotherapy technique, and induction chemotherapy. Given the limited number of local failure events, a sensitivity analysis using a reduced multivariable model was also performed, including FIGO stage, histology, HR-CTV D90, and radiotherapy technique. The associations between FIGO stage and external beam radiotherapy technique were assessed using the χ^2^ test. Statistical analyses were performed using Stata version 18 (StataCorp LLC, College Station, TX, USA). A two-sided *p*-value < 0.05 was considered statistically significant.

## 3. Results

Data from 267 patients who underwent radiotherapy between 2019 and 2021 were screened for inclusion. Of these, 30 patients were excluded: 8 without cervical cancer, 7 treated in the postoperative setting, 7 with metastatic disease, 4 with missing data, 2 with ineligible histology, and 2 treated with palliative intent. A total of 237 patients were included in the final analysis. The mean age was 58.2 ± 12.8 years, and 56.9% had FIGO stage III–IV disease. Squamous cell carcinoma was the predominant histological subtype, accounting for 83.5% of the cohort. Most patients received concurrent chemotherapy, with weekly cisplatin being the most common regimen. External beam radiotherapy was delivered primarily using conventional techniques in 184 patients (77.6%), while 53 patients (22.4%) received IMRT. At the fraction level, intracavitary brachytherapy was used more frequently than hybrid IC/IS brachytherapy, and the tandem–ovoid was the most commonly used applicator. Detailed patient, treatment, and dosimetric characteristics are summarized in Table 1.

**Figure 1:**
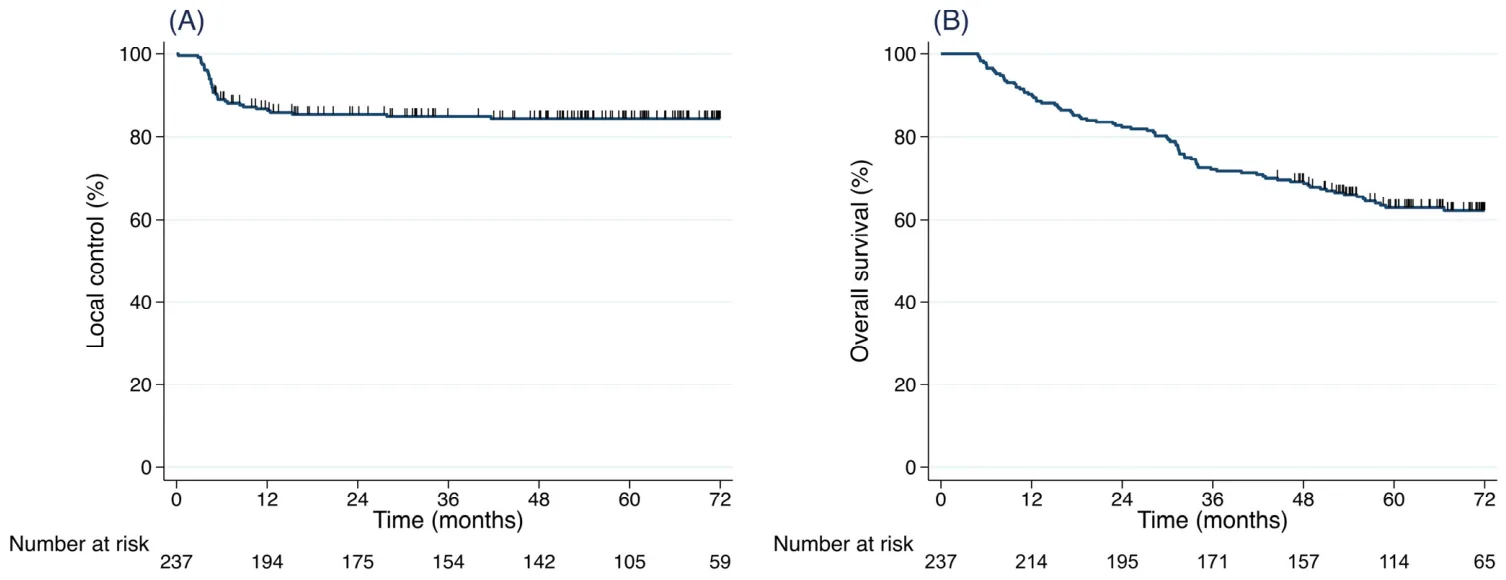
Kaplan–Meier curves for the entire cohort. (**A**) Local control. (**B**) Overall survival.

**Figure 2:**
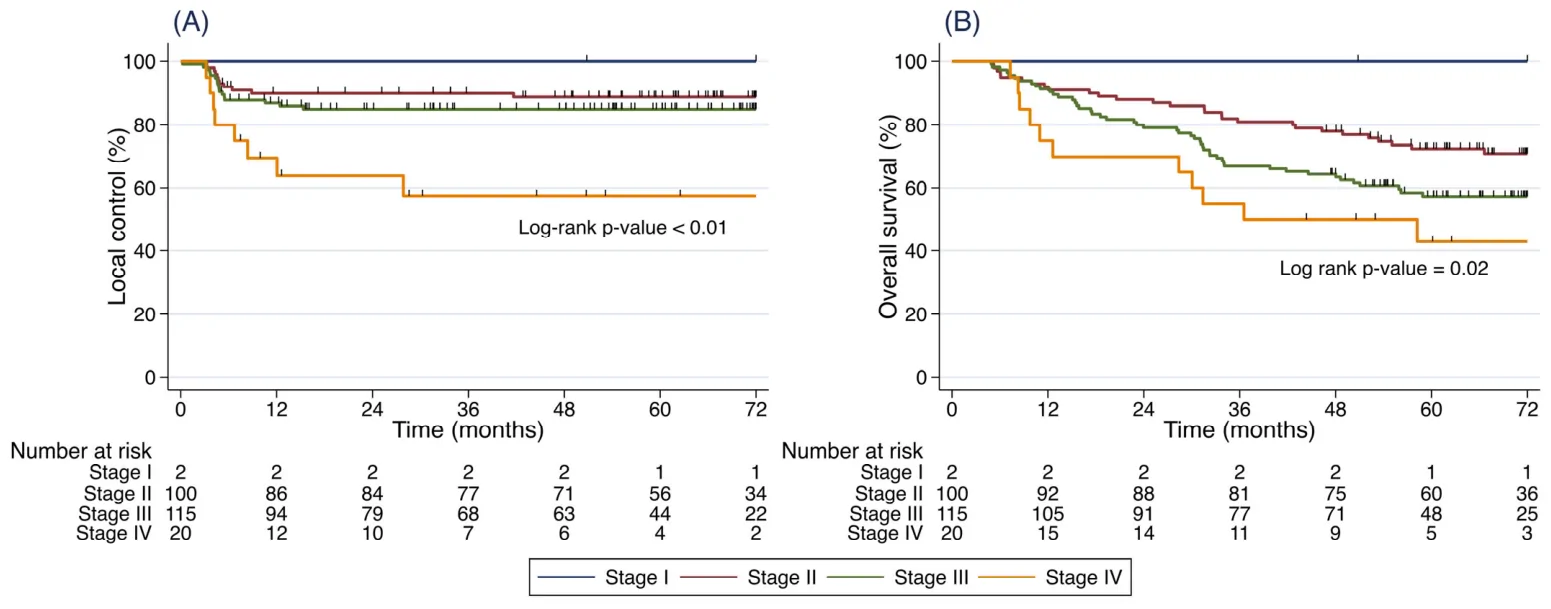
Kaplan–Meier curves stratified by FIGO 2018 stage. (**A**) Local control. (**B**) Overall survival.

At the six-week follow-up after completion of radiotherapy, 222 patients (93.7%) achieved a complete response, while 15 patients (6.3%) had persistent local disease. For the local control analysis, the median follow-up was 59.9 months (IQR, 19.5–71.1), during which 36 local failure events were observed. The 5-year local control rate was 84.4% (95% CI, 79.1–88.5%; Figure 1A). When death without prior local recurrence was treated as a competing event, the 5-year cumulative incidence of local recurrence was 15.2% (95% CI, 11.0–20.1%), corresponding to 84.8% remaining free from local recurrence. When stratified by FIGO 2018 stage, the 5-year local control rates were 100.0% for stage I, 88.8% (95% CI, 80.6–93.6%) for stage II, 85.0% (95% CI, 77.0–90.4%) for stage III, and 57.5% (95% CI, 32.2–76.3%) for stage IV. Local control differed significantly across stages (*p* < 0.01; Figure 2A). In the multivariable analysis, squamous histology and HR-CTV D90 ≥ 85 Gy EQD_2_ were independently associated with improved local control, whereas the use of IMRT was associated with poorer local control (Table 2). In a sensitivity analysis using a reduced model including FIGO stage, histology, HR-CTV D90, and radiotherapy technique, the findings remained consistent: squamous histology (HR 0.43, 95% CI 0.21–0.88; *p* = 0.02) and HR-CTV D90 ≥ 85 Gy EQD_2_ (HR 0.28, 95% CI 0.14–0.58; *p* < 0.01) were associated with improved local control, whereas IMRT (HR 3.50, 95% CI 1.76–6.96; *p* < 0.001) was associated with poorer local control.

For the overall survival analysis, the median follow-up was 71.1 months (IQR, 60.1–77.1), and the 5-year overall survival rate was 62.9% (95% CI, 56.3–68.8%; Figure 1B). When stratified by FIGO 2018 stage, the 5-year overall survival rates were 100.0% for stage I, 72.5% (95% CI, 62.5–80.3%) for stage II, 57.2% (95% CI, 47.4–65.9%) for stage III, and 42.9% (95% CI, 20.4–63.6%) for stage IV. Overall survival differed significantly across stages (*p* = 0.02; Figure 2B). In the multivariable analysis, squamous histology and receipt of ≥4 cycles of chemotherapy were independently associated with improved overall survival, whereas an overall treatment time > 56 days was independently associated with worse overall survival (Table 2).

To assess potential imbalance in disease stage between treatment groups, we compared the distribution of FIGO stage according to external beam radiotherapy technique. The stage distribution differed significantly between patients treated with IMRT and those treated with conventional radiotherapy (*p* = 0.002). Among the 53 patients treated with IMRT, none had stage I disease, 11 (20.8%) had stage II disease, 37 (69.8%) had stage III disease, and 5 (9.4%) had stage IV disease. Among the 184 patients treated with conventional radiotherapy, 2 (1.1%) had stage I disease, 89 (48.4%) had stage II disease, 78 (42.4%) had stage III disease, and 15 (8.2%) had stage IV disease.

In a sensitivity analysis restricted to patients with squamous cell carcinoma, the main prognostic patterns were generally consistent with the primary analysis. HR-CTV D90 ≥ 85 Gy EQD_2_ was associated with improved local control, while ≥4 chemotherapy cycles and shorter overall treatment time were associated with improved overall survival. IMRT was associated with poorer local control and overall survival in this subgroup.

## 4. Discussion

This retrospective cohort study demonstrated that routine CT-based IGABT achieved clinically meaningful outcomes among patients with cervical cancer. Among 237 patients treated between 2019 and 2021, the 5-year local control and overall survival rates were 84.4% and 62.9%, respectively.

HR-CTV D90 ≥ 85 Gy and squamous histology were independently associated with improved local control. Squamous histology and receipt of at least four cycles of chemotherapy were independently associated with improved overall survival, whereas overall treatment time > 56 days was associated with inferior overall survival. IMRT use was associated with poorer local control but was more common among patients with advanced-stage disease.

Compared with our previously reported cohort treated between 2008 and 2018, which had 4-year local control and overall survival rates of approximately 89% and 69%, respectively [16], the present cohort included a higher proportion of stage III–IV disease. However, comparisons between the two cohorts should be interpreted cautiously because of differences in follow-up duration and the transition to FIGO 2018 staging, which incorporates nodal status and may have resulted in stage migration.

Our findings support the real-world feasibility of implementing a structured CT-based IGABT program in settings where routine access to MRI-based IGABT, the preferred standard for image-guided cervical brachytherapy, remains limited. Our results suggest that well-implemented CT-based workflows can achieve outcomes broadly comparable to those reported in contemporary real-world brachytherapy series. These findings reinforce the practical role of CT-based IGABT as an achievable and scalable strategy for improving cervical cancer outcomes in settings with limited MRI access. This interpretation is supported by a recent study from India comparing MRI-based and CT-based IGABT, which demonstrated comparable outcomes after propensity score matching [20].

Although our local control and overall survival rates did not reach those reported in MRI-guided EMBRACE-I, the differences may partly reflect the higher proportion of stage III–IV disease in our cohort. The use of hybrid IC/IS techniques in our study (30.2%) remained lower than that in EMBRACE-I (43%) but increased substantially from 9% in our previous cohort [16]. In addition, our retrospective CT-based cohort reflects greater real-world heterogeneity in disease burden and healthcare service constraints than highly protocolized MRI-based programs. Nevertheless, our results are broadly aligned with those of the Retro-EMBRACE study [6], particularly in terms of overall survival. Furthermore, our findings are consistent with contemporary CT-based brachytherapy series from Thailand and Japan [21,22,23], further supporting the clinical feasibility and effectiveness of CT-based brachytherapy in routine practice where MRI availability remains limited (Table 3).

In multivariable analysis, squamous histology was independently associated with improved local control and overall survival. This finding is consistent with previous reports demonstrating inferior outcomes in adenocarcinoma and adenosquamous carcinoma compared with squamous cell carcinoma following definitive chemoradiotherapy [24,25].

Dose to the HR-CTV remained an important determinant of local control. In our cohort, 60.3% of patients achieved an HR-CTV D90 ≥ 85 Gy EQD_2_, which was independently associated with improved local control. This finding supports the established dose–response relationship reported in large image-guided brachytherapy series [26]. Although HR-CTV D90 was not independently associated with overall survival in the multivariable model, achieving an adequate target dose remains important for optimizing local disease control. Increasing the proportion of patients who achieve an HR-CTV D90 ≥ 85 Gy EQD_2_, with escalation toward 90 Gy EQD_2_ when clinically appropriate, may further improve local control.

Adequate chemotherapy delivery and timely treatment completion were also important. Receipt of at least four chemotherapy cycles was associated with improved overall survival, whereas overall treatment time > 56 days was associated with inferior survival. Although 70% of patients in our cohort completed treatment within 56 days, further efforts to minimize unnecessary delays may improve outcomes. These findings are consistent with previous evidence emphasizing the importance of adequate chemotherapy delivery and avoidance of unnecessary treatment delays during definitive chemoradiotherapy [27,28].

The observed association of IMRT with inferior local control should be interpreted cautiously, particularly as its association with overall survival was attenuated and no longer statistically significant after multivariable adjustment. IMRT was used more frequently in patients with advanced-stage or clinically complex disease, including those requiring extended-field or inguinofemoral irradiation. Thus, this association likely reflects confounding by indication and residual differences in disease burden. Current evidence indicates that IMRT reduces treatment-related toxicity while providing survival outcomes comparable to those achieved with conventional radiotherapy [29]. Therefore, our findings should not be interpreted as evidence of a detrimental effect of IMRT itself.

In the present cohort, the entire uterus was included in the CTV during EBRT. Emerging studies have explored individualized uterine EBRT, in which uninvolved portions of the uterine corpus are excluded from the target volume to reduce irradiation of surrounding normal tissues [30,31]. However, this approach relies on accurate assessment of the superior extent of disease. In predominantly CT-based settings such as ours, where routine MRI access is limited, accurate assessment of tumor extension into the uterine corpus may be challenging. Similar uncertainties apply to CT-based brachytherapy target delineation, particularly in defining the superior extent of residual disease, for which CT-based consensus recommendations advocate a more conservative target definition when MRI information is unavailable [32]. Therefore, although individualized uterine EBRT is an emerging approach of interest, its application in CT-dominant settings should be considered cautiously and requires further evaluation.

The strength of this study is that it reflects routine clinical practice, making the findings particularly relevant to institutions where MRI access is limited by cost, scheduling, or availability. The inclusion of a relatively large consecutive cohort and long-term outcome assessment further strengthens the reliability of the findings. Moreover, the high proportion of patients with advanced-stage disease provides clinically relevant evidence from a population often encountered in resource-limited settings. In such settings, CT-based brachytherapy represents a practical and scalable approach to improving cervical cancer care.

However, this study has several limitations. First, its retrospective design introduces the potential for selection and information bias. Second, the relatively small number of local failure events may limit the stability of the multivariable estimates for local control. However, the main findings remained unchanged in a sensitivity analysis using a reduced multivariable model. In addition, although Kaplan–Meier analysis may overestimate the probability of local recurrence when death is a competing event, a competing-risk analysis produced similar results. Third, the observed associations between external beam radiotherapy technique and clinical outcomes remain vulnerable to confounding by indication because IMRT was used more frequently in patients with advanced-stage or clinically complex disease. Fourth, treatment-related toxicity was not systematically evaluated, limiting assessment of the overall therapeutic benefit of this approach. Finally, the single-center design may limit the generalizability of the findings. Larger multicenter prospective studies are warranted to validate these results.

Future program development should focus on expanding the use of hybrid IC/IS brachytherapy, increasing the proportion of patients achieving an HR-CTV D90 ≥ 85 Gy EQD_2_, selectively escalating the dose toward 90 Gy EQD_2_ for large residual tumors, and improving treatment completion within 56 days. Further integration of transabdominal ultrasound guidance, optimized use of IMRT, and selective incorporation of MRI may also improve treatment quality. Emerging findings from the MRI-guided EMBRACE-II study support the potential benefits of these strategies within contemporary image-guided radiotherapy workflows [33].

## 5. Conclusions

Routine CT-based image-guided adaptive brachytherapy achieved clinically meaningful local control and overall survival despite a high proportion of stage III–IV disease. An HR-CTV D90 ≥ 85 Gy EQD_2_ was independently associated with improved local control, while receipt of at least four chemotherapy cycles and completion of treatment within 56 days were associated with improved overall survival. These findings support CT-based IGABT as a practical and scalable option for centers with limited access to MRI when delivered within a standardized adaptive brachytherapy workflow.

## Figures and Tables

**Table 1:** Patient, treatment, and dosimetric characteristics (*n* = 237).

Parameter	Value
Age (years), mean ± SD	58.2 ± 12.8
FIGO 2018 stage, *n* (%)	
I	2 (0.8)
II	100 (42.2)
III	115 (48.5)
IV	20 (8.4)
Histology, *n* (%)	
Squamous cell carcinoma	198 (83.5)
Adenocarcinoma	32 (13.5)
Adenosquamous carcinoma	7 (3.0)
Tumor size (cm), mean ± SD	4.9 ± 1.5
Tumor grade, *n* (%)	
Well-differentiated	17 (7.2)
Moderately differentiated	118 (49.8)
Poorly differentiated	57 (24.1)
Not reported	45 (19.0)
Imaging at diagnosis, *n* (%)	
CT	224 (94.5)
MRI	4 (1.7)
Not reported	9 (3.8)
Induction chemotherapy, *n* (%)	
No	199 (84.0)
Yes	38 (16.0)
Concurrent chemotherapy, *n* (%)	
None	26 (11.0)
Cisplatin	178 (75.1)
Carboplatin	33 (13.9)
External beam radiotherapy technique, *n* (%)	
IMRT	53 (22.4)
Conventional	184 (77.6)
Brachytherapy fractionation, *n* (%)	
7 Gy × 4 fractions	237 (100)
Brachytherapy approach, fractions, *n* (%)	
IC	660 (69.8)
Hybrid IC/IS	285 (30.2)
Applicator type, fractions, *n* (%)	
Tandem–ovoid	616 (65.2)
Tandem–ring	174 (18.4)
Tandem–cylinder	147 (15.6)
Other	8 (0.8)
Dosimetry (EQD_2_, Gy), mean ± SD	
HR-CTV D90	85.4 ± 2.7
Bladder D2cc	77.5 ± 5.1
Rectum D2cc	65.9 ± 5.7
Sigmoid D2cc	61.2 ± 9.9
HR-CTV volume at first fraction (cm^3^), median (IQR)	18.9 (13.4–28.4)
Overall treatment time (days), mean ± SD	53.1 ± 10.2

Abbreviations: SD = standard deviation; IQR = interquartile range; FIGO = International Federation of Gynecology and Obstetrics; CT = computed tomography; MRI = magnetic resonance imaging; IMRT = intensity-modulated radiation therapy; IC = intracavitary; IS = interstitial; HR-CTV = high-risk clinical target volume; D90 = minimum dose covering 90% of the high-risk clinical target volume; D2cc = minimum dose to the most exposed 2 cm^3^; EQD_2_ = equivalent dose in 2-Gy fractions.

**Table 2:** Univariable and multivariable Cox proportional hazards analyses for local control and overall survival.

Variable	Univariable HR (95% CI)	*p*-Value	Multivariable HR (95% CI)	*p*-Value
Local control				
Age > 58 years	1.05 (0.54–2.01)	0.89	1.27 (0.61–2.63)	0.53
FIGO stage III–IV (vs. I–II)	1.82 (0.90–3.70)	0.10	1.12 (0.47–2.68)	0.80
Squamous histology	0.42 (0.20–0.85)	0.02	0.41 (0.20–0.86)	0.02
Tumor size > 5 cm	1.74 (0.90–3.36)	0.10	1.40 (0.69–2.85)	0.35
HR-CTV D90 ≥ 85 Gy (EQD_2_)	0.25 (0.12–0.50)	<0.001	0.28 (0.13–0.57)	<0.01
≥4 chemotherapy cycles	0.85 (0.41–1.77)	0.67	0.97 (0.43–2.18)	0.94
Overall treatment time > 56 days	1.32 (0.66–2.65)	0.43	1.10 (0.53–2.29)	0.79
Lymph node metastasis	1.54 (0.77–3.08)	0.22	0.95 (0.40–2.27)	0.91
IMRT	4.23 (2.20–8.15)	<0.001	3.34 (1.60–6.97)	<0.01
Induction chemotherapy	1.80 (0.85–3.83)	0.13	1.52 (0.69–3.32)	0.30
Overall survival				
Age > 58 years	1.14 (0.75–1.73)	0.53	1.08 (0.69–1.67)	0.74
FIGO stage III–IV (vs. I–II)	1.75 (1.13–2.72)	0.01	1.40 (0.81–2.41)	0.23
Squamous histology	0.58 (0.36–0.95)	0.03	0.59 (0.35–0.98)	0.04
Tumor size > 5 cm	0.98 (0.63–1.54)	0.94	0.73 (0.45–1.19)	0.21
HR-CTV D90 ≥ 85 Gy (EQD_2_)	0.71 (0.47–1.08)	0.11	0.76 (0.49–1.17)	0.21
≥4 chemotherapy cycles	0.50 (0.32–0.76)	<0.01	0.61 (0.38–0.97)	0.04
Overall treatment time > 56 days	2.29 (1.50–3.49)	<0.001	2.12 (1.37–3.28)	<0.01
Lymph node metastasis	1.87 (1.21–2.90)	<0.01	1.36 (0.78–2.35)	0.28
IMRT	2.10 (1.35–3.27)	<0.01	1.61 (0.96–2.70)	0.07
Induction chemotherapy	1.27 (0.74–2.18)	0.39	1.11 (0.63–1.96)	0.72

Abbreviations: HR = hazard ratio; CI = confidence interval; FIGO = International Federation of Gynecology and Obstetrics; HR-CTV = high-risk clinical target volume; D90 = minimum dose covering 90% of the high-risk clinical target volume; EQD_2_ = equivalent dose in 2-Gy fractions; IMRT = intensity-modulated radiation therapy.

**Table 3:** Cross-study comparison of clinical characteristics and outcomes.

Characteristic	Retro-EMBRACE (FIGO 2009) [6]	EMBRACE-I (FIGO 2009) [7]	Murakami et al. (FIGO 2009) [21]	Uezono et al. (FIGO 2018) [22]	Dankulchai et al. (FIGO 2018) [23]	Our Study (FIGO 2018)
**Cohort size**	731	1341	469	171	354	237
**FIGO Stage**						
**I**	16.8%	18.1%	56.3% (I–II)	7%	3.4%	0.8%
II	55.9%	56.8%	—	20.3%	37.8%	42.2%
III	22.9%	15.2%	43.7% (III–IV)	72%	54.2%	48.5%
IV	3.1%	9.8%	—	2%	4.5%	8.4%
**IC/IS use**	23%	43%	40.3%	30%	37%	30.2%
**Local Control**	89% (5-year)	92% (5-year)	88.2% (4-year)	86% (3-year)	88% (5-year)	84.4% (5-year)
**Overall Survival**	65% (5-year)	74% (5-year)	83% (4-year)	75% (3-year)	61% (5-year)	62.9% (5-year)

Abbreviations: FIGO = International Federation of Gynecology and Obstetrics; IC/IS = combined intracavitary/interstitial brachytherapy.

## Data Availability

The data presented in this study are available from the corresponding author upon reasonable request. The data are not publicly available because of ethical and privacy restrictions concerning patient information.
